# A prospective cohort study on the association between neutrophil-to-lymphocyte and platelet-to-lymphocyte ratios and gestational diabetes mellitus in Chinese pregnant women

**DOI:** 10.3389/fendo.2025.1477092

**Published:** 2025-04-14

**Authors:** Xin Zhao, Jianbin Sun, Ning Yuan, Xiaomei Zhang

**Affiliations:** Department of Endocrinology, Peking University International Hospital, Beijing, China

**Keywords:** gestational diabetes mellitus, platelet-lymphocyte ratio, neutrophil-lymphocyte ratio, oral glucose tolerance test, glycosylated hemoglobin

## Abstract

**Aim:**

This study investigated whether neutrophil-to-lymphocyte ratio (NLR) and platelet-to-lymphocyte ratio (PLR) in early pregnancy correlate with subsequent development of gestational diabetes mellitus (GDM).

**Methods:**

This prospective cohort study enrolled 1,200 pregnant women during their first trimester at Peking University International Hospital between December 2017 and March 2019. All participants underwent oral glucose tolerance testing (OGTT) at 24-28 weeks gestation. Complete blood counts obtained in the first trimester were analyzed for NLR and PLR values. Participants were categorized into GDM (n=227) and non-GDM (n=973) groups based on International Association of Diabetes and Pregnancy Study Groups criteria.

**Results:**

Women who developed GDM exhibited significantly higher first-trimester levels of neutrophils, lymphocytes, platelets, NLR, and PLR (all p<0.05) compared to women without GDM. First-trimester NLR and PLR values positively correlated with second-trimester blood glucose levels at 0, 60, and 120 minutes during OGTT (all p<0.05). The optimal cut-off values for predicting GDM were 3.89 for NLR (sensitivity 76.05%, specificity 36.56%) and 148.11 for PLR (sensitivity 68.72%, specificity 68.65%). A multivariate predictive model incorporating NLR, PLR, age, parity, BMI, blood lipids, and uric acid demonstrated 78.39% sensitivity, 73.83% specificity, and 78.87% accuracy with an area under the curve of 0.79 (95% CI: 0.71, 0.86).

**Conclusions:**

First-trimester NLR and PLR represent independent risk factors for GDM development. These readily available inflammatory markers may have value for early GDM risk assessment and aid in targeting preventive interventions

## Introduction

Gestational diabetes mellitus (GDM) is a prevalent complication that results in detrimental health outcomes for both the mother and the infant (1). Studies have shown that GDM can cause adverse perinatal health effects, like macrosomia, preeclampsia, and neonatal hypoglycemia (2). Therefore, it is crucial to clinically prioritize the early detection and treatment of GDM, along with identifying the risk factors linked to its development (3).

Studies have demonstrated that the neutrophil-to-lymphocyte ratio (NLR) can indicate systemic inflammation (4). Moreover, the NLR is an indicator of the relative levels of neutrophils and lymphocytes in the body. This ratio is particularly relevant in chronic inflammatory diseases. A high NLR is indicative of the immune system’s functional status during chronic inflammation (5). Further, when compared to individual leukocyte parameters, the NLR demonstrates higher stability and is less susceptible to the effects of physiological, pathological, and physical factors. The NLR is a widely and inexpensive parameter that has been examined as a dependable potential marker of systemic inflammation in various chronic diseases (6). Current studies have demonstrated that the NLR is capable of evaluating the severity of many chronic diseases, such as chronic obstructive pulmonary disease (7), rheumatoid arthritis (8), sepsis (9), and Corona Virus Disease-19 (10), as well as multiple non-inflammatory conditions, including cerebrovascular disease (11), heart disease (12), diabetic macroangiopathy (13), diabetic nephropathy (DN) (14), and diabetic peripheral neuropathy (15). In the obstetric setting, the third-trimester NLR has been found elevated in pregnant women with pre-eclampsia (16) and has been associated with common carotid artery intimal-medial thickening in healthy pregnant women (17).

The platelet-to-lymphocyte ratio (PLR) has emerged as a novel inflammatory index that is preferred by researchers due to its rapidity, simplicity, and efficiency. Moreover, the combination of platelet (PLT) count with LYM absolute value can reflect the coagulation and immune response and the systemic inflammatory state of the body (18). Research on patients with type 2 diabetes mellitus (T2DM) demonstrated that the PLR increased substantially in patients with DN than in patients without T2DM. Further, the PLR was positively correlated with interleukin-6 and tumor necrosis factor-α levels (19). Therefore, the PLR can partially replace inflammatory indicators, reflect the body’s inflammatory level, and aid in the evaluation of clinically adverse effects in DN patients.

In both the maternal and fetal compartments, GDM is related to the disruption of multiple inflammatory mediators (20). However, the association between the PLR and NLR with GDM are conflicting. A study indicated that the increased NLR was a risk variable for GDM during initial pregnancy; however, no association was found between the PLR and GDM (21). More studies are needed to examine the correlation between the PLR and NLR with GDM.

While both NLR and PLR have demonstrated associations with inflammatory conditions, their relationship with GDM remains inconsistently reported in the literature. Previous studies have been limited by retrospective designs, small sample sizes, and varying diagnostic criteria for GDM. Furthermore, most investigations have focused on either NLR or PLR individually, without examining their combined predictive value. This prospective study addresses these limitations through a larger sample size, consistent diagnostic methodology, and comprehensive analysis of both markers.

Thus, this study was planned to examine the association between the PLR and NLR during the early stages of pregnancy and the formation of GDM to provide more compelling clinical evidence for the prevention of GDM.

## Materials and methods

### Study design

This study was carried out between December 2017 to March 2019. The Obstetrics Department of the Peking University International Hospital recruited pregnant women who were in their 1st trimester (7 to 12 gestation weeks). These women were consistently monitored to track the results of their pregnancies.

The study received approval from the bioethics committee of Peking University International Hospital (with approval number 2017-021). The protocols related to the ethical concerns were based on the institution and national committee, as well as the 1964 Declaration of Helsinki and its amendments. Each participant willingly agreed by providing written informed consent.

### Study population

1537 pregnant women participated in the study, specifically from the Obstetrics Department, between December 2017 and March 2019. Participants were recruited using a consecutive sampling approach during routine antenatal visits. All eligible pregnant women presenting for first-trimester care during the study period were invited to participate. The study was conducted at a single tertiary care center (Peking University International Hospital) which serves both urban Beijing residents and referrals from surrounding areas.

### Study criteria

Following the inclusion criteria were: (1) women who were ≥ 18 years; (2) who were willing to undergo the oral glucose tolerance test (OGTT) during 24 and 28 gestation weeks; (3) who were delivered at the hospital.

**Figure 1:**
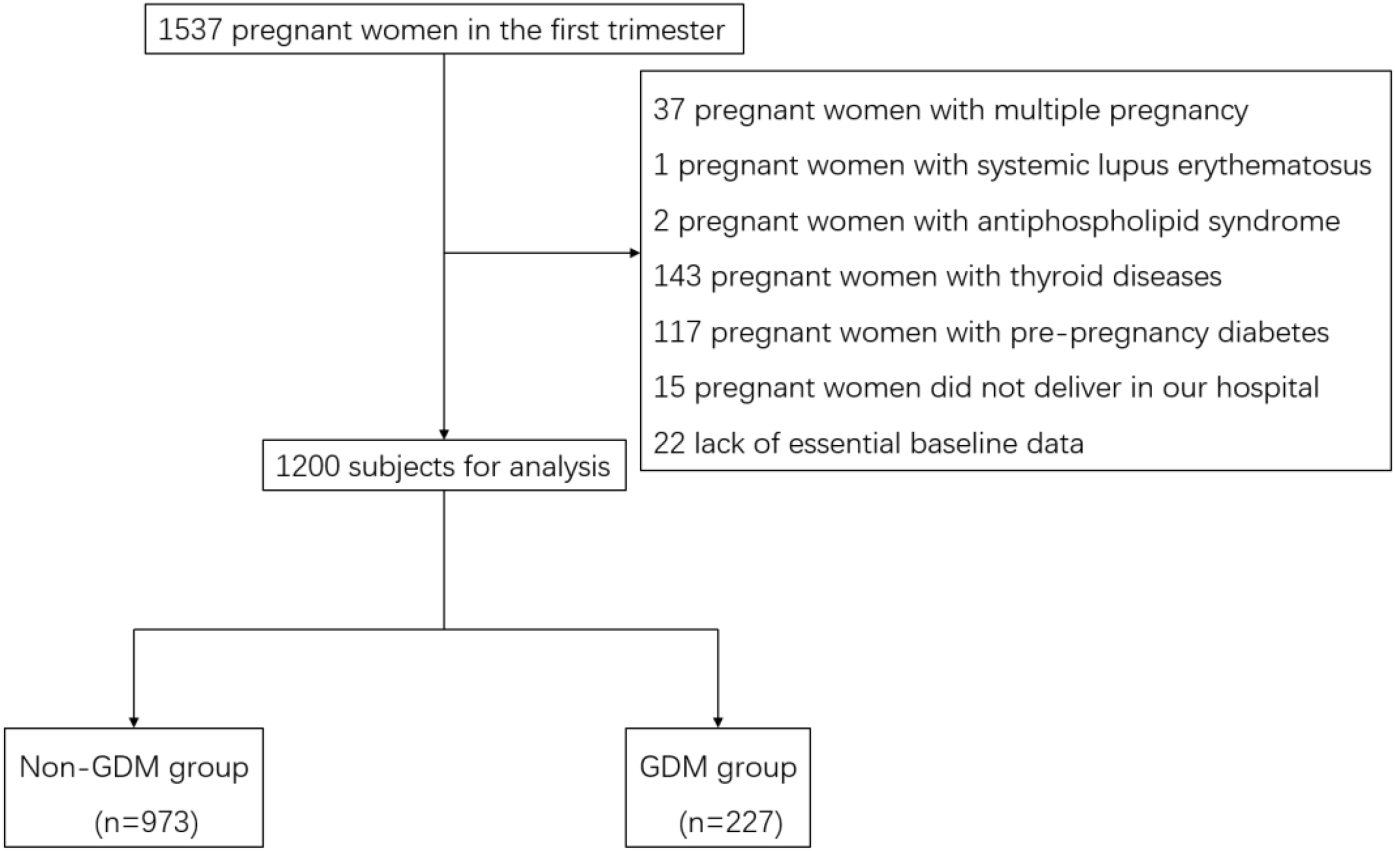
Flow chart of subject selection for the study. Flow chart showing the selection of study participants. Of 1,537 pregnant women initially assessed for eligibility, 337 were excluded for various reasons, resulting in a final study population of 1,200 participants (973 without GDM and 227 with GDM).

Following the exclusion criteria were: (1) detection of cardiovascular disease, thyroid disease, connective tissue disease, hematological disease, pulmonary disease or pre-pregnancy diabetes; (2) multiple pregnancies. (3) lack of essential baseline data. Finally, 1200 subjects with complete data were recruited in this study, calculated to detect an effect size of 0.5 with 85% power at a significance level of 0.05 in a two-tailed independent t-test (**Figure 1**).

### General information

During participation, basic information was collected such as age, parity, and personal history of GDM, systolic blood pressure (SBP), diastolic blood pressure (DBP), as well as weight, height, and body mass index (BMI). The BMI was measured *via* the formula: BMI (kg/m^2^) = weight (kg)/body height^2^ (m^2^). The average age of 1200 pregnant women was 30.91 ± 3.68 years old, the proportion of multiparous women was 41.75%, the average BMI was 21.97 ± 3.04 kg/m^2^, the average SBP was 109.99 ± 10.58mmHg, and the average DBP was 66.16 ± 8.94 mmHg.

This study followed up with pregnant women until delivery and recorded the occurrence of adverse pregnancy outcomes including premature rupture of membranes, placental abruption, premature delivery, fetal distress, macrosomia, and low birth weight. Adverse pregnancy outcomes were defined using the following standardized criteria: premature rupture of membranes (spontaneous rupture of membranes before the onset of labor); placental abruption (premature separation of the placenta from the uterine wall); premature delivery (delivery before 37 completed weeks of gestation); fetal distress (abnormal fetal heart rate pattern suggesting fetal hypoxia, requiring intervention); macrosomia (birth weight >4000g); and low birth weight (birth weight <2500g).

These outcomes were documented by obstetricians at delivery and confirmed through medical record review.

### Laboratory index detection

During the 7–12 weeks of gestation, a venous blood sample (5 mL) was drawn from all participants in the morning while they were fasting. The following were the detection indexes: NEUT, LYM, platelet (PLT), fasting blood glucose (FBG), C-reactive protein (CRP), total cholesterol (TC), glycosylated hemoglobin (HbA1c), high-density lipoprotein cholesterol (HDL-C), triglycerides (TG), uric acid (UA), serum creatinine (SCr) and low-density lipoprotein cholesterol (LDL-C) levels. Simultaneously, the PLR and NLR were determined. High-performance liquid chromatography was conducted to record the HbA1c levels *via* a Dongcao G8 analyzer. FBG,TC,LDL-C,HDL-C, TG, UA and SCr were conducted using Chemiluminescence method. NEUT, LYM and PLT were conducted using Flow impedance method. CRP was conducted using Enzyme-linked Immuno- Sorbent Assay method. The average HbA1c was 5.24 ± 1.21% and the average FBG was 4.90 ± 0.39mmol/L. The average NEUT was (5.74 ± 1.72)10^9^/L, the average LYM was (1.87 ± 0.73) 10^9^/L, and the average PLT was (242.40 ± 53.18) 10^9^/L.

### Detection of GDM

All women were examined for GDM by conducting the OGTT (75 g) during 24 to 28 weeks of gestation. They were hospitalized in the morning and were recommended to fast for 8 to 12 h before OGTT. They were orally administered 75 g of an anhydrous powder mixed for 5 min in 250 to 300 mL of warm boiled water. The level of blood glucose was recorded before (GLU_0min_), 1 h (GLU_60min_), and 2 h (GLU_120min_) after drinking the glucose mixture.

The International Association of Diabetes and Pregnancy Study Groups (IADPSG) diagnostic criteria were followed for the detection of GDM (22). According to IADPSG criteria, GDM was diagnosed when one or more of the following thresholds were met or exceeded during the 75g OGTT: fasting plasma glucose ≥5.1 mmol/L, 1-hour glucose ≥10.0 mmol/L, or 2-hour glucose ≥8.5 mmol/L. These criteria were chosen as they have been validated in Chinese populations and are currently recommended by Chinese clinical practice guidelines.

During the second trimester, weight, height, and BMI were collected. The gestational weights gain during the second trimester were calculated and recorded.

These women were assigned to the GDM (n = 227) and non-GDM (n = 973) groups. The correlation between the clinical data of these expectant women during their 1st trimester and the development of GDM was also explored.

### Statistical analysis

Data were analyzed using SPSS version 22.0 (IBM Corp., Armonk, NY, USA). Normality of continuous variables was assessed using the Kolmogorov-Smirnov test. Normally distributed data were presented as mean ± standard deviation and compared using independent-samples t-tests. Non-normally distributed data were presented as median (interquartile range) and compared using Mann-Whitney U tests. Categorical variables were expressed as frequencies (percentages) and compared using chi-square or Fisher’s exact tests as appropriate.

Correlations between laboratory parameters and OGTT values were evaluated using Spearman’s rank correlation coefficient. Univariate logistic regression was first performed to identify potential predictors of GDM. Variables with p<0.10 in univariate analysis were entered into multivariate logistic regression models to calculate adjusted odds ratios (OR) and 95% confidence intervals (CI). NLR and PLR were categorized into tertiles (low, middle, high) based on the distribution in the non-GDM group to assess dose-response relationships.

Receiver operating characteristic (ROC) curves were constructed to evaluate the predictive value of NLR and PLR for GDM development. The Youden index (sensitivity + specificity - 1) was used to determine optimal cut-off values. A multivariate predictive model was developed incorporating clinical variables and inflammatory markers, and its performance was assessed through area under the curve (AUC),sensitivity, specificity, and accuracy calculations.

For analysis of adverse pregnancy outcomes, we used logistic regression models to evaluate associations between first-trimester NLR/PLR values and each outcome, adjusting for known risk factors. We also calculated the relative risks (RR) and odds ratios (OR) with 95% confidence intervals for each adverse outcome comparing GDM and non-GDM groups. Mediation analysis was performed to determine whether associations between inflammatory markers and adverse outcomes were mediated through GDM. All statistical tests were two-sided, with p<0.05 considered statistically significant.

## Results

### Comparison of basic and laboratory indexes between groups in 1st trimester and OGTT findings

Out of the 1200 pregnant women, 227 were screened with GDM in the 2nd trimester, leading to a 23.08% prevalence rate. The observed GDM prevalence of 23.08% is higher than typically reported in general obstetric populations. This may reflect the use of IADPSG criteria, which are known to increase GDM diagnosis rates compared to older diagnostic standards. Additionally, our tertiary hospital setting may have resulted in a patient population with more risk factors for GDM than community-based samples. We analyzed demographic and clinical characteristics to identify potential explanatory factors for this prevalence rate. The GDM group had a substantially higher number of multiparous women (χ^2^ = 9.44, *p* < 0.05) in contrast to the non-GDM group. The gestational weight gain was considerably higher in the GDM group in comparison to the non-GDM group (*p* < 0.05). In the initial trimester, women with GDM manifested increased BMI, HbA1c, and FBG levels than those without GDM (*p* < 0.05). Moreover, the GDM group revealed markedly increased levels of CRP, TC, TG, LDL-C, and UA than the non-GDM group (all *p <* 0.05). Similarly, the NLR, the PLR, and the NEUT, LYM, and PLT counts were all increased in the GDM group in comparison to the non-GDM group (all *p <* 0.05). The incidence of macrosomia in the GDM group was significantly higher than that in the non-GDM group (*p* < 0.05), and there was no statistically significant difference in other adverse pregnancy outcomes (all *p* > 0.05). To explore potential influencing factors on inflammatory markers, we conducted subgroup analyses stratifying participants by age (<30 vs. ≥30 years), pre-pregnancy BMI (<24 vs. ≥24 kg/m²), and parity (nulliparous vs. multiparous). The association between elevated NLR/PLR and GDM remained significant across all subgroups, with slightly stronger associations observed in women with higher BMI (interaction p=0.042) but no significant interaction with age or parity (interaction p=0.38 and p=0.57, respectively) (**Table 1**).

**Table 1:** Comparison of baseline characteristics and laboratory parameters between women who developed GDM and those who remained normoglycemic.

Index	Non-GDM group	GDM group	t (X^2^)	p-value
	(n=973)	(n=227)	value	
Maternal characteristics				
Age (years)	31.54 ± 2.61	30.27 ± 3.16	0.73	0.50
BMI (kg/m^2^)	21.45 ± 3.09	23.34 ± 3.90	-7.62	<0.05
**Parity, n (%)**			9.44	<0.05
Nulliparous	588 (60.43)	111 (48.90)		
Multiparous	385 (39.57)	116 (51.10)		
SBP (mmHg)	111.06 ± 10.46	109.71 ± 10.26	0.43	0.69
DBP (mmHg)	66.44 ± 8.90	64.70 ± 8.58	2.60	<0.05
Metabolic parameters				
TC (mmol/L)	3.90 ± 0.68	4.01 ± 0.60	-2.60	<0.05
TG (mmol/L)	0.95 ± 0.48	1.10 ± 0.30	-4.06	<0.05
LDL-C (mmol/L)	2.02 ± 0.50	2.11 ± 0.45	-2.18	<0.05
HDL-C (mmol/L)	1.31 ± 0.28	1.32 ± 0.27	-0.41	0.61
UA (umol/L)	231.92 ± 45.54	229.80 ± 47.67	-4.67	<0.05
sCr (umol/L)	48.47 ± 7.01	48.98 ± 6.78	1.13	0.19
HbA1c (%)	5.18 ± 0.20	5.29 ± 0.27	-5.42	<0.05
FBG (mmol/L)	4.87 ± 0.39	5.04 ± 0.41	-6.09	<0.05
Gestational weight gain (kg)	9.24 ± 1.23	11.99 ± 2.32	-5.03	<0.05
CRP (mg/L)	1.98 ± 2.69	3.04 ± 3.21	-5.49	<0.05
Inflammatory markers				
NEUT (x10^9^/L)	5.68 ± 1.68	6.00 ± 1.83	-2.59	<0.05
LYM (x 10^9^/L)	1.84 ± 0.45	1.94 ± 0.47	-2.74	<0.05
PLT (x 10^9^/L)	240.25 ± 52.54	252.96 ± 52.85	-3.28	<0.05
NLR	3.03 ± 1.13	3.24 ± 1.21	-2.01	<0.05
PLR	127.12 ± 41.25	136.65 ± 39.80	-1.96	<0.05
OGTT results				
Fasting glucose (mmol/L)	4.49 ± 0.29	4.96 ± 0.57	-17.44	<0.05
1-hour glucose (mmol/L)	7.31 ± 1.31	9.71 ± 1.63	-23.71	<0.05
2-hour glucose (mmol/L)	6.61 ± 0.97	8.47 ± 1.64	-22.60	<0.05
Adverse pregnancy outcomes, n (%)				
Premature rupture of membranes	158 (18.10)	26 (11.45)	0.99	0.32
Placental abruption	14 (1.60)	3 (1.32)	0.01	0.91
Fetal distress	72 (8.25)	13 (5.73)	0.12	0.73
Macrosomia	51 (5.84)	24 (10.57)	6.23	<0.05
Low birth weight	8 (0.92)	4 (1.76)	0.72	0.40
Premature delivery	45 (5.15)	13 (5.73)	0.01	0.99

Values are presented as mean ± standard deviation or number (percentage).

BMI, body mass index; SBP, systolic blood pressure; DBP, diastolic blood pressure; FBG, fasting blood glucose; HbA1c, glycosylated hemoglobin; UA, uric acid; TC, total cholesterol; TG, triglycerides; LDL-C, low-density lipoprotein cholesterol; HDL-C, high-density lipoprotein cholesterol; NEUT, neutrophil count; LYM, lymphocyte count; PLT, platelet count; NLR, neutrophil-to-lymphocyte ratio; PLR, platelet-to-lymphocyte ratio; OGTT, oral glucose tolerance test; CRP, C-reactive protein; sCr, serum creatinine.

### Association between inflammatory markers, GDM and adverse pregnancy outcomes

Women who developed GDM had a significantly higher incidence of macrosomia compared to those without GDM (10.57% vs. 5.84%, p<0.05). No significant differences were observed between groups for other adverse outcomes including premature rupture of membranes (11.45% vs. 18.10%, p=0.32), placental abruption (1.32% vs. 1.60%, p=0.91), fetal distress (5.73% vs. 8.25%, p=0.73), low birth weight (1.76% vs. 0.92%, p=0.40), and premature delivery (5.73% vs. 5.15%, p=0.99) (**Table 1**).

We further analyzed the relationship between first-trimester inflammatory markers and adverse pregnancy outcomes. After adjusting for maternal age, BMI, and parity, women in the highest tertile of NLR had increased odds of macrosomia (adjusted OR 1.92, 95% CI 1.18-3.11, p=0.009) compared to those in the lowest tertile. This association remained significant after further adjustment for GDM status (adjusted OR 1.78, 95% CI 1.07-2.95, p=0.027), suggesting that the relationship between NLR and macrosomia is partially independent of GDM development. PLR was not independently associated with any adverse pregnancy outcomes after adjustment for confounders.

Mediation analysis revealed that GDM mediated approximately 23% of the association between first-trimester NLR and macrosomia (indirect effect p=0.038), while the direct effect of NLR on macrosomia remained significant. Neither NLR nor PLR showed significant associations with other adverse outcomes in adjusted models(**Table 2**).

**Table 2:** Association between first-trimester inflammatory markers and adverse pregnancy outcomes.

Outcome	Overall incidence (%)	NLR tertiles			Adjust OR* (95%CI)	p-value	Mediation by GDM (%)
		Lowest	Middle	Highest			
Macrosomia	75 (6.25)	17 (4.25)	23 (5.75)	35 (8.75)	1.92 (1.18-3.11)	0.009	23% (p-0.038)
Premature rupture of membranes	184 (15.33)	63 (15.75)	59 (14.75)	62 (15.50)	1.12 (0.75-1.67)	0.578	
Placental abruption	17 (1.42)	5 (1.25)	6 (1.50)	6 (1.50)	1.23 (0.37-4.11)	0.736	
Fetal distress	85 (7.08)	29 (7.25)	27 (6.75)	29 (7.25)	0.97 (0.57-1.67)	0.922	
Low birth weight	12 (1.00)	4 (1.00)	3 (0.75)	5 (1.25)	1.31 (0.35-4.92)	0.686	
Premature delivery	58 (4.83)	17 (4.25)	19 (4.75)	22 (5.50)	1.38 (0.72-2.65)	0.328	

*Adjusted for maternal age, pre-pregnancy BMI, parity, and GDM status.

NLR, neutrophil-to-lymphocyte ratio; OR, odds ratio; CI, conﬁdence interval; GDM, gestational diabetes mellitus.

Tertile cut-points for NLR were: lowest (≤2.55), middle (2.56-3.42), and highest (>3.42).

### Laboratory indexes and blood glucose association with and without glucose loading

In the 1st trimester, BMI, TG, TC, LDL-C, and UA levels were strongly linked with blood glucose concentration with and without glucose dosing in the 2nd trimester (*p* < 0.05). The weight gain was also positively related to blood glucose levels in the 2nd trimester in the same glucose dosing condition (*p* < 0.05). Furthermore, the NLR, PLR, NEUT, LYM, and PLT levels in the 1st trimester were all closely linked with blood glucose concentrations in the same glucose loading condition in the 2nd trimester (all *p* < 0.05) (**Tables 3**, **4**).

**Table 3:** Correlation analysis between laboratory indexes and blood glucose before and after glucose loading.

Index	GLU_0min_		GLU_60min_		GLU_120min_	
	r	p	r	p	r	p
BMI (kg/m^2^)	0.25	<0.05	0.17	<0.05	0.26	<0.05
SBP (mmHg)	-0.06	0.06	-0.07	<0.05	-0.03	0.38
DBP (mmHg)	-0.10	<0.05	-0.10	<0.05	-0.07	<0.05
TC (mmol/L)	0.09	<0.05	0.13	<0.05	0.13	<0.05
TG (mmol/L)	0.15	<0.05	0.15	<0.05	0.15	<0.05
LDL-C (mmol/L)	0.13	<0.05	0.12	<0.05	0.12	<0.05
HDL-C (mmol/L)	-0.11	<0.05	-0.01	0.84	-0.04	0.14
UA (umol/L)	0.16	<0.05	0.14	<0.05	0.16	<0.05
sCr (umol/L)	-0.02	0.54	-0.01	0.70	-0.03	0.30
gestational weight gain (kg)	0.09	<0.05	0.04	0.09	0.11	<0.05

BMI is for body mass index, SBP is systolic blood pressure, DBP is for diastolic blood pressure, FBG is for fasting blood glucose, HbA1c is for glycosylated hemoglobin, sCr is for serum creatinine, UA is for uric acid, TC is for total cholesterol, TG is for triglycerides, LDL-C is for low-density lipoprotein cholesterol, HDL-C is for high-density lipoprotein cholesterol, GLU0min is for fasting blood glucose before OGTT, GLU60min is for blood glucose 60 min after OGTT, GLU120min is for blood glucose 120 min after OGTT.

**Table 4:** Correlation Analysis between NLR and PLR and blood glucose before and after glucose loading.

Index	GLU_0min_		GLU_60min_		GLU_120min_	
	r	p	r	p	r	p
NEUT	0.09	<0.05	0.12	<0.05	0.13	<0.05
LYM	0.06	0.05	0.09	<0.05	0.06	0.05
PLT	0.07	<0.05	0.08	<0.05	0.07	<0.05
NLR	0.16	<0.05	0.07	<0.05	0.08	<0.05
PLR	0.19	<0.05	0.17	<0.05	0.21	<0.05

NEUT is for neutrophil, LYM is for lymphocyte, PLT is for platelet, NLR is for neutrophil to lymphocyte ratio, PLR is for platelet to lymphocyte ratio, GLU0min is for fasting blood glucose before OGTT, GLU60min is for blood glucose 60 min after OGTT, GLU120min is for blood glucose 120 min after OGTT.

### Logistic regression analysis of the PLR, NLR, and GDM

A multivariate logistic regression analysis was carried out, with GDM as the dependent factor and important variables identified from the univariate analysis as the independent factors. After controlling for age, parity, BMI, blood cholesterol level, BP, and UA and SCr levels, the PLR and NLR were identified as distinct risk factors for GDM (**Table 5**).

**Table 5:** Univariate and multivariate logistic regression analyses of the association between first-trimester inflammatory markers and GDM development.

Variable	Category	Crude OR (95%CI)	p-value	Adjust OR* (95%CI)	p-value
PLR					
	Lowest tertile	1.00 (Reference)		1.00 (Reference)	
	Middle tertile	1.00 (0.69,1.46)	0.88	0.97 (0.66,1.44)	0.86
	High tertile	1.57 (1.11,2.23)	<0.05	1.46 (1.03,2.12)	<0.05
NLR					
	Lowest tertile	1.00 (Reference)		1.00 (Reference)	
	Middle tertile	2.34 (1.45,3.78)	<0.05	2.29 (1.38,3.81)	<0.05
	High tertile	7.60 (4.89,11.82)	<0.05	7.97 (4.98,12.76)	<0.05

*Adjusted for age, parity, pre-pregnancy BMI, blood pressure, lipid proﬁle, and uric acid levels.

OR, odds ratio; CI, conﬁdence interval; PLR, platelet-to-lymphocyte ratio; NLR, neutrophil-to-lymphocyte ratio; GDM, gestational diabetes mellitus.

### Single variable predicting model of GDM

**Figure 2:**
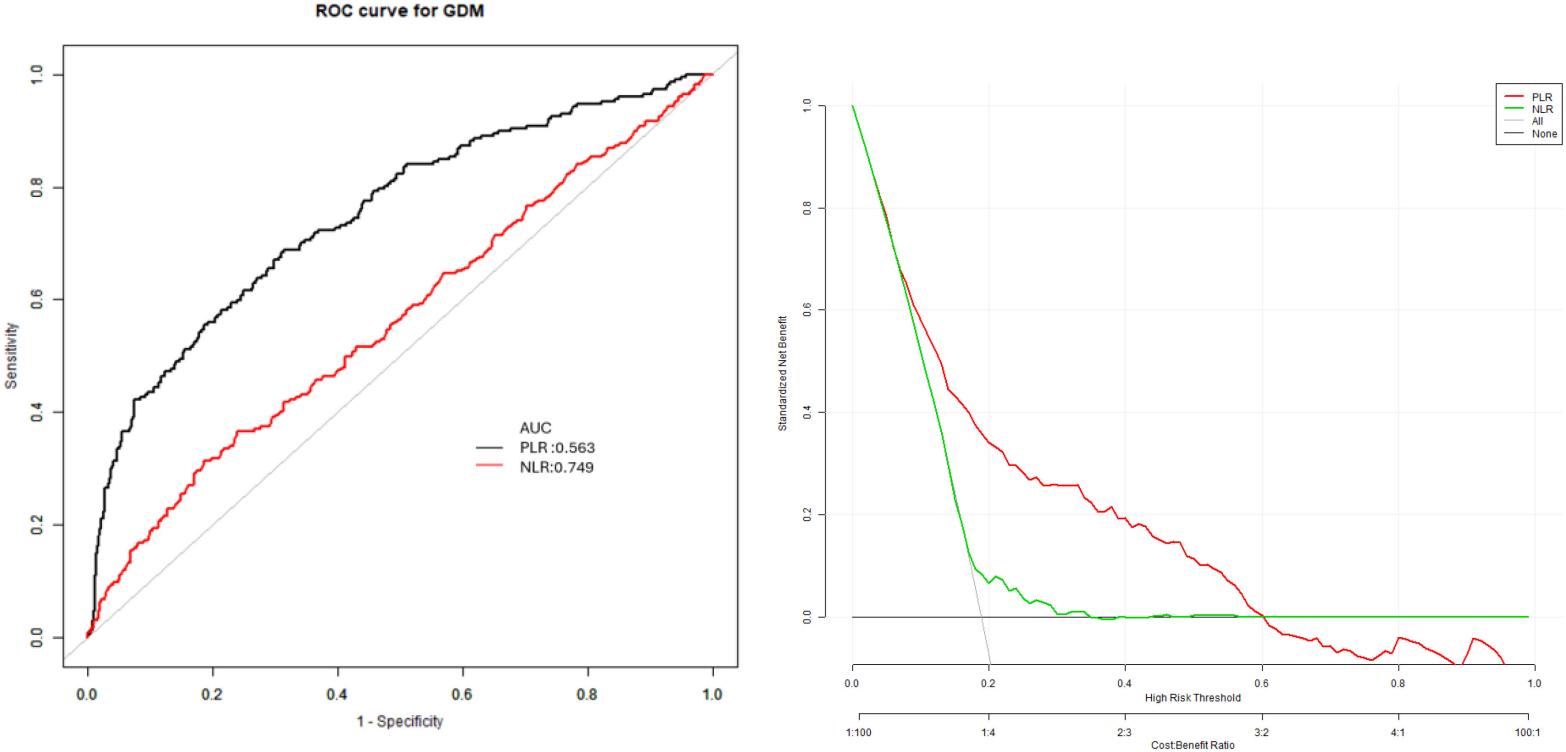
Receiver operating characteristic (ROC) curves for the prediction of gestational diabetes mellitus using neutrophil-to-lymphocyte ratio (NLR) and platelet-to-lymphocyte ratio (PLR). ROC curves showing the predictive performance of first-trimester NLR and PLR for GDM. The area under the curve (AUC) was 0.75 (95% CI: 0.71, 0.78) for NLR and 0.57 (95% CI: 0.52, 0.61) for PLR.The optimal cut-off values were 3.89 for NLR (sensitivity 76.05%, specificity 36.56%) and 148.11 for PLR (sensitivity 68.72%, specificity 68.65%).

In the predicting model of GDM, which included specific factors like the PLR and NLR, their cut-off values were 3.89 and 148.11, respectively (**Figure 2**; **Table 6**).

**Table 6:** Univariate predictive models of GDM with NLR and PLR.

Index	AUC (95%CI)	Specificity	Sensitivity	Cut-off
PLR	0.57 (0.52, 0.61)	36.56%	76.05%	3.89
NLR	0.75 (0.71, 0.78)	68.65%	68.72%	148.11

NLR is for neutrophil-to-lymphocyte ratio, PLR is for platelet-to-lymphocyte ratio.

**Figure 3:**
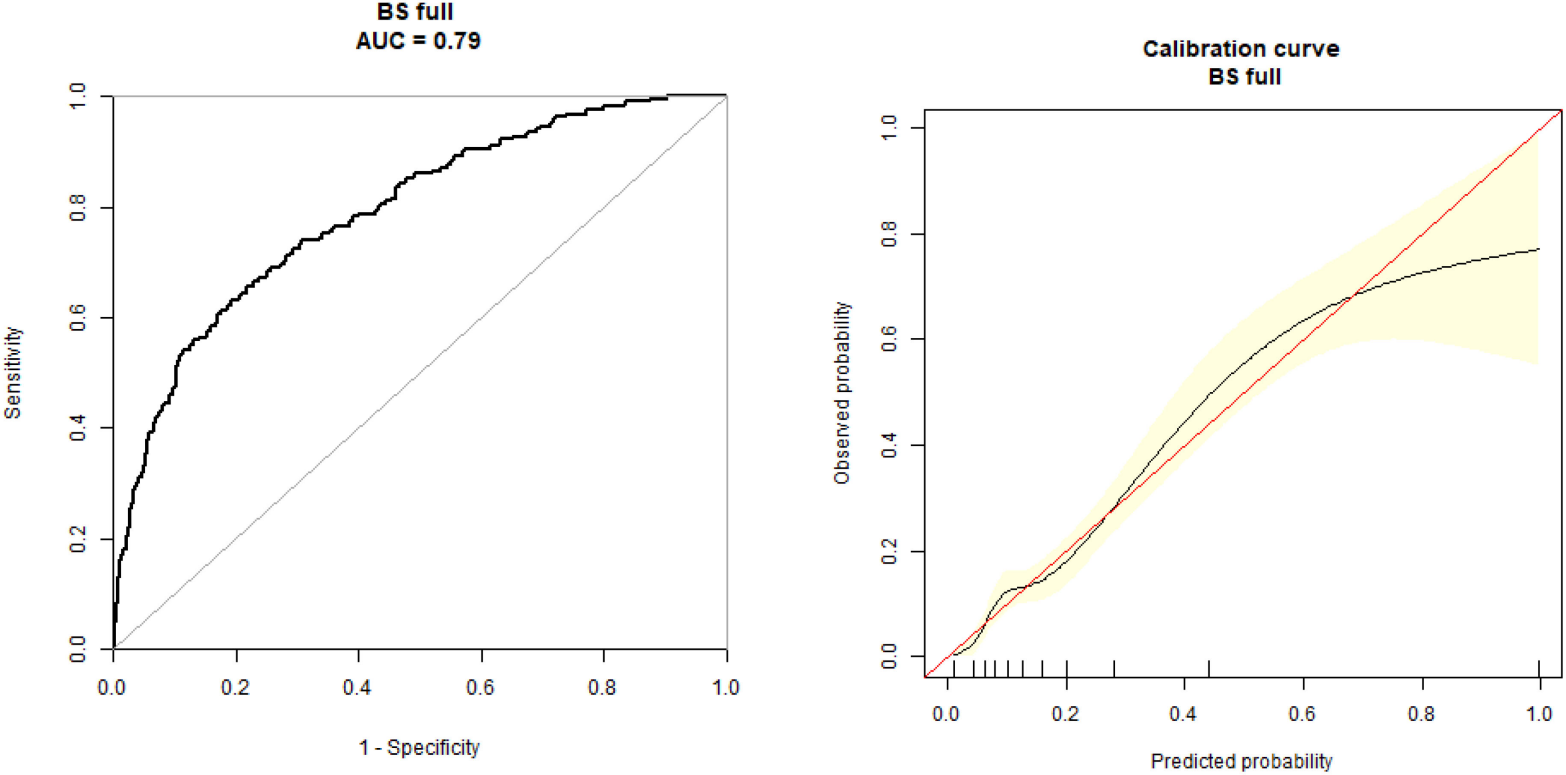
Receiver operating characteristic (ROC) curve for the multivariate prediction model of gestational diabetes mellitus. ROC curve for the multivariate model incorporating NLR, PLR, age, parity, BMI, blood lipids, and uric acid levels for the prediction of GDM. The model achieved an area under the curve of 0.79 (95% CI: 0.71, 0.86) with a sensitivity of 78.39%, specificity of 73.83%, and accuracy of 78.87%.

The dependent factor in the multivariate predictive model was GDM, while the independent factors were age, parity, BMI, blood lipid, BP, UA, PLR, and NLR levels. The specificity (73.83%), sensitivity (78.39%), and accuracy (78.87%), values were observed with AUC (0.79) (95% CI: 0.71, 0.86) (**Figure 3**).

## Discussion

GDM leads to significant perinatal problems, such as macrosomia, cesarean section, shoulder dystocia, and neonatal hypoglycemia. It has also prolonged effects on the development of T2DM in mothers and obesity in children (2). The GDM prevalence in our cohort (23.08%) was considerably higher than the 10-18% typically reported in Chinese populations using IADPSG criteria. This elevated rate likely reflects several factors: (1) the use of IADPSG diagnostic criteria, which are known to increase GDM diagnosis by 2-3 fold compared to older Carpenter-Coustan criteria; (2) the tertiary hospital setting, which may attract higher-risk pregnancies;(3) increasing maternal age and BMI in urban Chinese populations; and (4) the comprehensive screening approach that captured mild cases that might be missed in routine care. Previous studies from similar urban Chinese settings have reported GDM rates ranging from 17.5% to 19.7% using IADPSG criteria, suggesting our finding is within the expected range for this population and diagnostic approach, albeit at the higher end. The relationship between inflammation and target organ damage in diabetes (particularly T2DM), is intricate. For example, hypoxia-activated adipocytes secrete cytokines and adipokines, which may act as pro-inflammatory factors (23). Excess glucose levels in diabetic patients can disrupt the body’s natural hemostasis and induce the secretion of pro-inflammatory cells and mediators.

The NLR is a new potential marker of inflammation. It may be elevated by neutrophilia or lymphopenia. Moreover, this index can be readily accessed *via* appropriate blood tests. Research has indicated a correlation between the NLR and endocrine and metabolic diseases (24), suggesting that inflammation is a major factor in many chronic diseases. The NLR is a systemic marker of inflammation that is significantly involved in the identification of short and long-term survival of heart and cancer patients (25). The NLR shows a comparable association with diabetic microvascular effects, such as DNP and diabetic retinopathy (16, 26). The primary cause of blindness in adults with diabetes is diabetic retinopathy, and an increase in the NLR is a critical indicator for its diagnosis (27). The patients with T2DM are in a high glucose environment for a long time due to the increased level of advanced glycation end products. Inflammatory factors such as C-reactive protein, tumor necrosis factor alpha, and interleukin 6 are easy to activate. All these factors may cause the increase of neutrophil count. At this stage, patients exhibit innate immune defense abnormalities, and their neutrophil chemotaxis, phagocytosis, and ability to kill bacteria are all damaged to varying degrees (28).

In a clinical study that included 5620 T2DM patients (29), 3374 diabetic patients experienced at least one microvascular problem, while the remaining 2246 patients did not manifest any microvascular complications. The NLR of patients with diabetes who experienced at least one microvascular complication was 1.14 times higher (*p* ≤ 0.01) than that of patients with diabetes who did not have any complications. Consequently, the NLR is a low-cost, efficient, and readily available inflammatory marker that has the potential to serve as a vital indicator of microvascular problems in T2DM patients. The PLR is a unique inflammatory factor that has recently attracted attention due to its ability to predict various circulation-related diseases and conditions, including myocardial infarction, reperfusion disorder post percutaneous coronary intervention, malignancy, rheumatic disease progression, and inflammation. It increases when the platelet count increases and the lymphocyte count decreases, which is associated with an unfavorable cardiovascular event prognosis and increased inflammation (18). The increased platelet count interferes with blood microcirculation in the kidney, thereby interrupting the blood flow and aggravating hypoxia in the kidney (18).

The association between inflammatory markers and GDM continues to yield inconsistent results across studies (30). Pace et al. conducted a meta-analysis of 11 studies (n=1,271) and found a significant association between elevated NLR and GDM development, consistent with our findings (31). However, their analysis was limited by small sample sizes and methodological heterogeneity. Sargın et al. reported that first-trimester NLR, but not PLR, predicted GDM in Turkish women (n=228), partially aligning with our results (32). In contrast, Hessami et al. found no association between first-trimester PLR and GDM in their meta-analysis, contradicting our observation of PLR as an independent risk factor (21).

These discrepancies may be explained by several factors. First, different diagnostic criteria for GDM were employed across studies, with some using two-step Carpenter-Coustan criteria while others, including ours, used one-step IADPSG criteria which identifies milder cases of glucose intolerance. Second, the timing of inflammatory marker measurement varied, with some studies collecting samples at GDM diagnosis rather than in early pregnancy. Third, ethnic differences in inflammatory profiles and GDM pathophysiology may contribute to variable findings, as inflammatory responses and insulin resistance patterns differ across populations. Finally, sample size limitations in previous studies may have reduced statistical power to detect associations, particularly for PLR which demonstrated a weaker correlation than NLR in our analysis. In another recent study, 110 expectant women were examined to determine the association between GDM and many inflammatory markers, such as WBCs and PLT counts, mean PLT volume, PLT crit, the NLR, and the PLR. The results indicated that there were no substantial intergroup differences in the NEUT and LYM counts, as well as the NLR (33). It was challenging to determine whether the inflammatory markers in the early stages of pregnancy corresponded with the development of GDM due to the small sample size and the determination of inflammatory indicator levels at 24–28 weeks in the 2nd trimester.

Our findings indicate that both NLR and PLR have potential value as early pregnancy screening tools for GDM. The predictive accuracy of NLR (AUC 0.75) was moderate and superior to that of PLR (AUC 0.57), suggesting NLR may be more clinically useful as an individual marker. However, neither marker alone achieved the predictive accuracy necessary for clinical implementation as a standalone screening test. When combined with established risk factors in our multivariate model, predictive performance improved (AUC 0.79), offering promise for enhanced risk stratification in early pregnancy. While these results suggest potential clinical utility, prospective validation in diverse populations is necessary before clinical implementation can be recommended.

Our analysis of adverse pregnancy outcomes revealed that women with GDM had a significantly higher incidence of macrosomia, consistent with established literature documenting the association between maternal hyperglycemia and excessive fetal growth. Interestingly, we found that first-trimester NLR was independently associated with macrosomia even after adjusting for GDM status, suggesting that maternal inflammation may contribute to fetal overgrowth through pathways distinct from glucose metabolism. Mediation analysis indicated that approximately 23% of the association between elevated NLR and macrosomia was mediated through GDM, while the majority of the effect was direct or through alternative pathways.

This finding aligns with emerging evidence that maternal inflammation may affect placental function and nutrient transfer independent of glycemic control. Systemic inflammation has been linked to altered placental gene expression, vascular development, and transport function, potentially leading to dysregulated fetal growth. The lack of association between PLR and adverse pregnancy outcomes in our study suggests that neutrophil-driven inflammation may have more specific efflects on maternal-fetal exchange than platelet activation.

The absence of significant associations between inflammatory markers and other adverse outcomes, such as premature rupture of membranes or preterm delivery, differs from some previous reports. This discrepancy may reflect our study’s focus on early pregnancy inflammatory status rather than inflammation at the time of delivery, or the relatively low incidence of certain complications in our cohort, limiting statistical power to detect modest associations.

For predictive model development, GDM was employed as a dependent factor, while age, parity, BP, BMI, blood lipid, HbA1c, UA, NLR, and PLR levels were all used as independent factors. The specificity (73.83%), sensitivity (78.39%), and accuracy (78.87%) values of the prediction model indicate its predictive value for the early detection of GDM. The AUC of this model was 0.79 (95% CI: 0.71, 0.86).

This study has several limitations that should be acknowledged. First, despite its prospective design, our study was conducted at a single tertiary care center, potentially limiting generalizability to community-based populations. The relatively high GDM prevalence in our cohort (23.08%) may reflect referral bias and limit applicability to lower-risk settings. Second, we measured inflammatory markers only once during the first trimester, preventing assessment of how these values change throughout pregnancy. Longitudinal measurements might provide insight into the dynamic relationship between inflammatory markers and glucose metabolism during pregnancy. Third, while we adjusted for multiple confounders, residual confounding from unmeasured variables cannot be excluded. Factors such as dietary patterns, physical activity, family history of diabetes, and genetic predisposition may influence both inflammatory markers and GDM risk but were not comprehensively assessed in our study. Fourth, our prediction model was developed and tested in the same population without external validation. The reported predictive accuracy may be optimistic, and validation in independent cohorts is essential before clinical application. Fifth, we did not have pre-pregnancy inflammatory marker measurements for comparison, making it difficult to distinguish pregnancy-induced changes from pre-existing inflammatory status. Sixth, we lacked detailed data on maternal infections or other acute inflammatory conditions at the time of blood sampling, which could have transiently elevated inflammatory markers independent of metabolic risk. Seventh, we did not measure other inflammatory markers like high-sensitivity C-reactive protein, interleukin-6, or TNF-α, which might provide additional insights into the inflammatory pathways linking NLR/PLR to GDM pathogenesis. Finally, while we documented several adverse pregnancy outcomes, our study was not primarily powered to detect associations between inflammatory markers and these relatively rare events, particularly placental abruption and low birth weight.

## Conclusion

In conclusion, the association between the NLR and PLR and GDM in expectant women was analyzed, revealing that the PLR and NLR in the 1st trimester were distinct risk factors for GDM with exceptionally high predictive values for the development of GDM. Therefore, both of these markers may offer novel evidence that can aid in the early detection of GDM.

## Data Availability

The original contributions presented in the study are included in the article/supplementary material. Further inquiries can be directed to the corresponding author.
